# DG-LSTM-SA model: A deep gated LSTM network with self-attention mechanism for power generation and load forecasting

**DOI:** 10.1371/journal.pone.0350071

**Published:** 2026-06-03

**Authors:** Guoqiang Sun, Yang Zhao, Jianglong Li, Junfu Cui, Xiaoyan Qi

**Affiliations:** 1 Naval Aviation University, Qingdao, People’s Republic of China; 2 School of Information Science and Engineering, Shandong University, Qingdao, People’s Republic of China; 3 Department of Biochemistry and Molecular Biology, Shandong Medical and Pharmaceutical University, Yantai, People’s Republic of China; Shahid Beheshti University, IRAN, ISLAMIC REPUBLIC OF

## Abstract

Accurate forecasting of power generation and load demand is essential for the reliable operation of modern energy systems. Traditional recurrent neural networks (RNNs) often struggle to capture long-term dependencies in complex power time series, whereas recent Transformer-based models can introduce substantial computational overhead. To address these limitations, we propose a Deep Gated Long Short-Term Memory network with Self-Attention (DG-LSTM-SA). The proposed model combines a multi-layer gated architecture with hierarchically embedded self-attention modules, enabling it to adaptively emphasize informative time steps and capture complex temporal patterns without a prohibitive increase in parameters. We evaluated DG-LSTM-SA on three real-world energy datasets (NEPOOL, Yichang, and Solar-Energy). The results demonstrate that DG-LSTM-SA consistently outperforms ten baseline models. Compared with standard RNN variants such as LSTM and GRU, DG-LSTM-SA substantially reduces forecasting errors, decreasing Mean Absolute Error by more than 75%. Furthermore, relative to state-of-the-art attention-based models (e.g., Informer and Crossformer), DG-LSTM-SA achieves competitive accuracy while maintaining a distinct advantage in computational efficiency and training speed. Comprehensive ablation studies further confirm that the proposed design is robust, accurate, and practical for real-world grid dispatch and operational decision-making.

## 1. Introduction

As global energy demand increases and renewable energy integration expands, power systems have become more complex and uncertain [1,2]. Accurate forecasting of power generation and load demand is therefore critical for maintaining system stability and reliability [3–5]. Reliable forecasts help grid operators anticipate fluctuations in generation and consumption, supporting decisions on resource allocation and grid management [6–9]. Traditional statistical approaches, such as autoregressive integrated moving average (ARIMA) [10] and exponential smoothing [11], often have limited capability in modeling long-term dependencies and non-linear dynamics. Consequently, machine learning and deep learning methods, including regression trees [12] and support vector machines [13], have been increasingly adopted to capture complex temporal patterns in modern energy systems [14,15]. Recent studies further show that deep data-driven models can substantially outperform traditional approaches in challenging forecasting scenarios [16,17].

In deep learning, sequence-to-sequence modeling has long been dominated by Recurrent Neural Networks (RNNs) and their variants [18,19]. Long Short-Term Memory (LSTM) and Gated Recurrent Unit (GRU) networks improve basic RNNs through gating mechanisms that regulate information flow [20,21]. To further enhance sequential modeling, several advanced RNN architectures have been proposed. For instance, Independently RNN (IndRNN) [22] alleviates gradient issues by decoupling neuron interactions across layers, while Skip RNN (SKIP-RNN) [23] improves efficiency by skipping redundant state updates. More recently, enhanced architectures such as LSTM-g [24] and extended LSTM (xLSTM) [25] introduce exponential gating strategies and expanded memory structures. Despite these advances, a key limitation of the RNN family remains their strictly sequential processing and heavy reliance on hidden states to summarize historical context. This structure makes it challenging for them to adaptively emphasize distant yet important time steps, a common requirement in volatile real-world energy datasets [26].

Attention mechanisms, particularly self-attention, have reshaped time-series forecasting by enabling flexible dependency modeling [27]. The Transformer architecture [28], originally developed for natural language processing, replaces recurrence with global self-attention. To improve long-sequence forecasting, optimized Transformer variants have been proposed, such as Informer [29] with ProbSparse attention and Crossformer [30] with cross-dimensional dependency modeling. However, Transformer-based approaches can also be suboptimal for energy forecasting. Without the sequential inductive bias of recurrence, Transformers often often rely heavily on positional encodings to represent temporal order [31]. Furthermore, stacking multiple attention layers can lead to high computational cost and large parameter size, which limits training efficiency and real-time deployment in grid operations [29–31].

**Fig 1:**
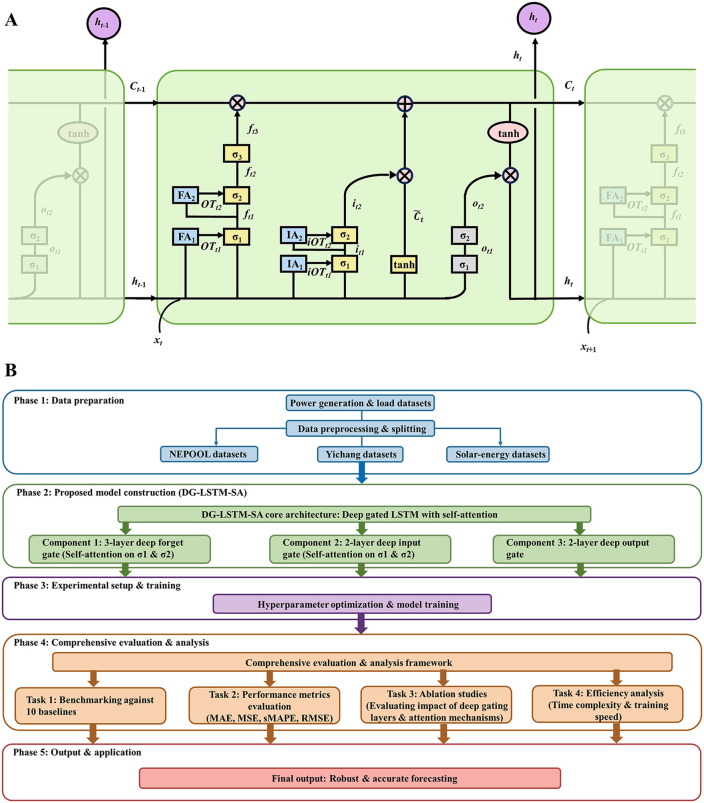
(A) Schematic of the DG-LSTM-SA model for power generation and load forecasting. **(B)** Flowchart of this study.

To address the limitations of both RNN and Transformers, this study bridges deep-gated recurrence and hierarchical dynamic attention [32,33]. We propose a hybrid architecture, named Deep Gated LSTM with Self-Attention (DG-LSTM-SA) (Fig 1A). Instead of applying global self-attention across all time steps, DG-LSTM-SA embeds self-attention modules within the internal multi-layered gates of an LSTM. This design preserves the sequential inductive bias of RNNs while enabling the gating mechanisms selectively emphasize critical historical information, improving accuracy without excessive computational overhead.

The main contributions are as follows:

(1) We propose DG-LSTM-SA, a novel and efficient architecture that addresses the limitations of standard RNNs and Transformers, achieving superior forecasting accuracy on power generation and load tasks.
(2) We conduct systematic optimization and ablation studies to quantify the the contributions of deep hierarchical gating with gate-level self-attention.
(3) We benchmark DG-LSTM-SA against ten state-of-the-art baselines (RNN, LSTM, GRU, IndRNN, SKIP-RNN, LSTM-g, xLSTM, Transformer, Informer, and Crossformer) on three real-world datasets, demonstrating advantages in both forecasting accuracy and computational efficiency.

The remainder of this paper is organized as follows. Sect 2 presents the DG-LSTM-SA methodology. Sect 3 describes datasets, experimental settings, and evaluation metrics. Sect 4 presents the experimental results, including optimization, comparisons, and ablation studies. Sect 5 concludes the study and outlines future directions. A workflow overview is provided in Fig 1B.

## 2. Methodology

The DG-LSTM-SA architecture is illustrated in Fig 1A. The model consists of deep forget, input, and output gating mechanisms integrated with hierarchical self-attention modules. Specifically, the forget gate uses three sigmoid layers ($\sigma_{\text{1}}$, $\sigma_{\text{2}}$, and $\sigma_{\text{3}}$) to generate $f_{t\text{1}}$, $f_{t\text{2}}$, and $f_{t\text{3}}$, and embeds attention modules ${\text{FA}}_{\text{1}}$ and ${\text{FA}}_{\text{2}}$ to produce attention outputs ${OT}_{t\text{1}}$ and ${OT}_{t\text{2}}$. Similarly, the input gate uses two sigmoid layers $\sigma_{\text{1}}$ and $\sigma_{\text{2}}$ to compute $i_{t\text{1}}$ and $i_{t\text{2}}$, guided by attention modules ${\text{IA}}_{\text{1}}$ and ${\text{IA}}_{\text{2}}$ that output ${iOT}_{t\text{1}}$ and ${iOT}_{t\text{2}}$. The output gate is designed as a two-layer structure. Detailed formulations are given below.

### 2.1 Deep forget gated mechanism with self-attention

**Fig 2:**
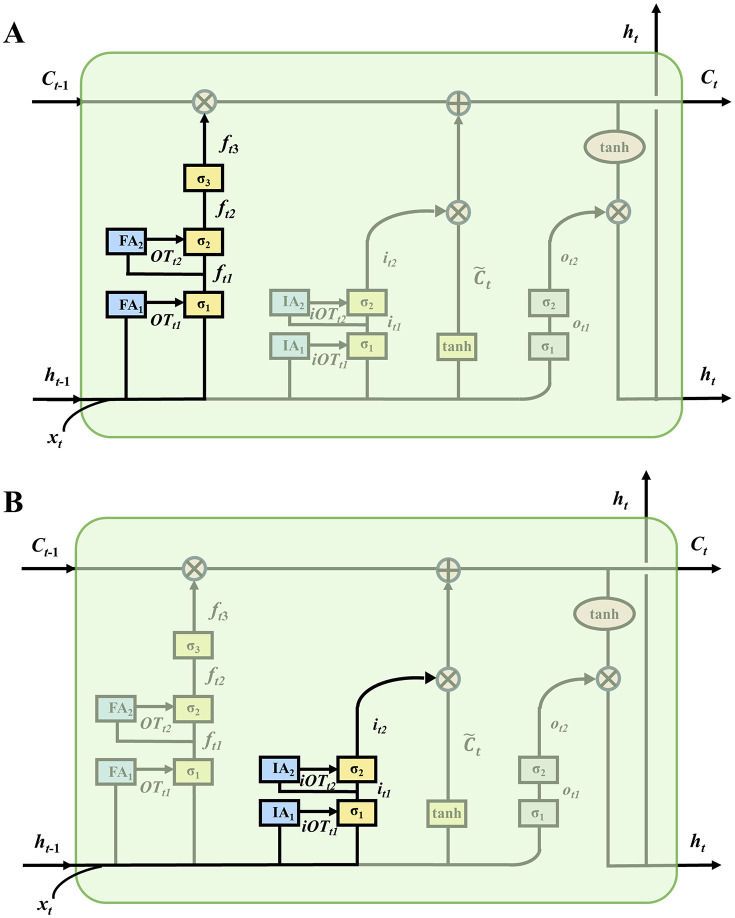
Schematic of the three-layer forget gate with attention mechanism (A) and two-layer input gate with attention mechanism (B) in the DG-LSTM-SA model.


As shown in Fig 2A, the standard single-layer LSTM forget gate is extended to a three-layer structure, as described by Eqs (1), (2), and (3):

$$\begin{array}{c} f_{t\text{1}}=\sigma_{\text{1}}(W_{f\text{1}}\cdot [h_{t-\text{1}},x_{t}]+b_{f\text{1}}) \end{array}$$ (1)

$$\begin{array}{c} f_{t\text{2}}=\sigma_{\text{2}}(W_{f\text{2}}\cdot f_{t\text{1}}+b_{f\text{2}}) \end{array}$$ (2)

$$\begin{array}{c} f_{t\text{3}}=\sigma_{\text{3}}(W_{f\text{3}}\cdot f_{t\text{2}}+b_{f\text{3}}) \end{array}$$ (3)

where $f_{t\text{1}}$, $f_{t\text{2}}$, and $f_{t\text{3}}$ represent the outputs of the first, second, and third layers of the forget gate, respectively, and · denotes matrix multiplication. The first layer, $f_{t\text{1}}$ is computed similarly to a standard LSTM forget gate, using the input $x_{t}$, the previous hidden state $h_{t-\text{1}}$, weight matrix $W_{f\text{1}}$, and bias $b_{f\text{1}}$, followed by a sigmoid function $\sigma_{\text{1}}$. Unlike in a standard LSTM, $f_{t\text{1}}$ does not directly interact with the previous cell state $C_{t-\text{1}}$; instead, it is passed to the second gate layer.

In the second layer, $f_{t\text{1}}$ is processed with weight matrix $W_{f\text{2}}$, and bias $b_{f\text{2}}$, followed by a sigmoid function $\sigma_{\text{2}}$ to yield $f_{t\text{2}}$. This process is repeated in the third layer with $f_{t\text{2}}$, producing the final output $f_{t\text{3}}$, which selectively forgets components of the cell state $C_{t-\text{1}}$, similar to a standard LSTM forget gate.

For $f_{t\text{1}}$, the sequence ${AXf}^{\left(\text{1}\right)}\;$= [$h_{t-\text{1}};x_{t}$] is processed through an attention mechanism ${\text{FA}}_{\text{1}}$ with queries, keys, and values computed as Eqs (4), (5), and (6):


$$\begin{array}{c} Q_{f\text{1}}={AXf}^{\left(\text{1}\right)}\;W^{Qf\text{1}} \end{array}$$ (4)

$$\begin{array}{c} K_{f\text{1}}={AXf}^{\left(\text{1}\right)}\;W^{kf\text{1}} \end{array}$$ (5)

$$\begin{array}{c} V_{f\text{1}}={AXf}^{\left(\text{1}\right)}\;W^{vf\text{1}} \end{array}$$ (6)

where $W^{Qf\text{1}}$, $W^{kf\text{1}}$ and $W^{vf\text{1}}$ are the weight matrices for the query, key, and value of $f_{t\text{1}}$, respectively, while $Q_{f\text{1}}$, $K_{f\text{1}}$ and $V_{f\text{1}}$ represent their corresponding query, key, and value matrices.

Next, the similarity between $Q_{f\text{1}}$ and $K_{f\text{1}}$ is calculated, followed by the application of a softmax function to obtain the attention weights as expressed in Eq (7):


$$\begin{array}{c} A_{f\text{1}}=\text{softmax}(\frac{Q_{f\text{1}}\;{K_{f\text{1}}}^{T}}{\sqrt{d_{kf\text{1}}}}) \end{array}$$ (7)

where $Q_{f\text{1}}\;{K_{f\text{1}}}^{T}$ denotes the product of the query matrix and the transpose of the key matrix. $d_{kf\text{1}}$ represents the dimensionality of the key vectors. $A_{f\text{1}}$ is the attention weight matrix.

The attention weight matrix $A_{f\text{1}}$ corresponds to each element $\overset{\left(\text{1}\right)}{\underset{i}{AXf}}$ in the sequence ${AXf}^{\left(\text{1}\right)}$. Each row of the matrix $A_{f\text{1}}$ is then multiplied by the columns of the matrix $V_{f\text{1}}$ through a dot-product operation, resulting in an output vector ${OT}_{{t\text{1}}_{i}}$ for each element i in the sequence, as described in Eq (8):


$$\begin{array}{c} {OT}_{{t\text{1}}_{i}}=\sum_{j=\text{1}}^{n}{A_{f\text{1}}}_{ij}{V_{f\text{1}}}_{j} \end{array}$$ (8)

where n denotes the sequence length of the temporal input. ${A_{f\text{1}}}_{ij}$ is the element in the i-th row and j-th column of the matrix $A_{f\text{1}}$, representing the attention weight of the element $\overset{\left(\text{1}\right)}{\underset{i}{AXf}}$ towards the element $\overset{\left(\text{1}\right)}{\underset{j}{AXf}}$. ${V_{f\text{1}}}_{j}$ denotes the j th vector in $V_{f\text{1}}$.

By aggregating the output vectors ${OT}_{{t\text{1}}_{i}}$ of all elements, the final output matrix ${OT}_{t\text{1}}=[{OT}_{{t\text{1}}_{\text{1}}};{OT}_{{t\text{1}}_{\text{2}}};\ldots ;{OT}_{{t\text{1}}_{n}}]$ is formed. The final output, obtained by performing a weighted sum on the values $V_{f\text{1}}$ using the attention weights, can be represented as Eq (9):


$$\begin{array}{c} {OT}_{t\text{1}}=A_{f\text{1}}\;V_{f\text{1}}. \end{array}$$ (9)

Meanwhile, the attention weights for the second forget gate $f_{t\text{2}}$ is denoted as ${OT}_{t\text{2}}$. Its weight uses $f_{t\text{1}}$ as the input sequence for its attention mechanism ${\text{FA}}_{\text{2}}$, i.e., ${AXf}^{\left(\text{2}\right)}$=$f_{t\text{1}}$. For each element $\overset{\left(\text{2}\right)}{\underset{i}{AXf}}$ in the sequence ${AXf}^{\left(\text{2}\right)}$, it is mapped to query, key, and value vectors using three different weight matrices $Q_{f\text{2}}$, $K_{f\text{2}}$, and $V_{f\text{2}}$.

Subsequently, to enable the model to effectively forget non-essential or redundant information, ${OT}_{t\text{1}}$and ${OT}_{t\text{2}}$ are introduced into $f_{t\text{1}}$ and $f_{t\text{2}}$, respectively (Fig 2A). Specifically, $f_{t\text{1}}$ integrates ${OT}_{t\text{1}}$, $x_{t}$, and $h_{t-\text{1}}$ and then, together with ${OT}_{t\text{2}}$, passes to the $\sigma_{\text{2}}$ unit to compute $f_{t\text{2}}$. Then, $f_{t\text{2}}$ is processed by $\sigma_{\text{3}}$ to generate the final forget gate $f_{t\text{3}}$. Ultimately, $f_{t\text{3}}$ interacts with $C_{t-\text{1}}$ to determine which features from $C_{t-\text{1}}$ are retained for the computation of $C_{t}$, as described in Eqs (10), (11), and (12):


$$\begin{array}{c} f_{t\text{1}}=\sigma_{\text{1}}(W_{f\text{1}}\cdot [h_{t-\text{1}},x_{t},\;{OT}_{t\text{1}}]+b_{f\text{1}}) \end{array}$$ (10)

$$\begin{array}{c} f_{t\text{2}}=\sigma_{\text{2}}(W_{f\text{2}}\cdot {[f}_{t\text{1}},\;{OT}_{t\text{2}}]+b_{f\text{2}}) \end{array}$$ (11)

$$\begin{array}{c} f_{t\text{3}}=\sigma_{\text{3}}(W_{f\text{3}}\cdot f_{t\text{2}}+b_{f\text{3}}) \end{array}$$ (12)

### 2.2 Deep input gated mechanism with self-attention

As shown in Fig 2B, the input gate $i_{tn}(\text{n}\in [\text{1},\text{2}])$ in the deep input gating mechanism has a similar two-layer structure as the forget gate. The first layer of the input gate is responsible for the preliminary screening of input features, determining the importance of information from the basic inputs $h_{t-\text{1}}$ and $x_{t}$. This process can be described as Eq (13):


$$\begin{array}{c} i_{t\text{1}}=\sigma_{\text{1}}(W_{i\text{1}}\cdot [h_{t-\text{1}},x_{t}]+b_{i\text{1}}) \end{array}$$ (13)

where $i_{t\text{1}}$ represents the first layer of the input gate, calculated from the input data $x_{t}$ of the current DG-LSTM-SA unit and the hidden state $h_{t-\text{1}}$ passed from the previous unit. This calculation involves applying a weight matrix $W_{i\text{1}}$, adding a bias term $b_{i\text{1}}$, and then processing the result through a sigmoid function $\sigma_{\text{1}}$.

After the first layer input gate $i_{t\text{1}}$ is computed, the result is passed directly to the second layer input gate. There, it is computed with the second weight matrix $W_{i\text{2}}$, and the result is summed with the bias $b_{i\text{2}}$, followed by another sigmoid function to obtain the second layer input gate $i_{t\text{2}}$. This process is described by Eq (14):


$$\begin{array}{c} i_{t\text{2}}=\sigma_{\text{2}}(W_{i\text{2}}\cdot i_{t\text{1}}+b_{i\text{2}}) \end{array}$$ (14)

Additionally, by introducing attention mechanisms ${\text{ IA}}_{\text{1}}$ and ${\text{IA}}_{\text{2}}$ within the two-layer input gates, the model’s ability to select input features is further enhanced, thereby improving the DG-LSTM-SA’s capacity to capture long-term dependencies (Fig 2B). For the first layer input gate $i_{t\text{1}}$, the input sequence for its attention mechanism is ${AXi}^{\left(\text{1}\right)}$=[$h_{t-\text{1}};x_{t}$], where t represents the current time step.

First, each element ${AXi}^{\left(\text{1}\right)}$ in the input sequence $\overset{\left(\text{1}\right)}{\underset{i}{AXi}}$ is encoded using three different weight matrices $Q_{i\text{1}}$, $\;K_{i\text{1}}$ and $V_{i\text{1}}$, as shown in Eqs (15), (16), and (17):


$$\begin{array}{c} Q_{i\text{1}}={AXi}^{\left(\text{1}\right)}\;W^{Qi\text{1}}+\overset{\left(\text{1}\right)}{\underset{q}{bi}} \end{array}$$ (15)

$$\begin{array}{c} K_{i\text{1}}={AXi}^{\left(\text{1}\right)}\;W^{ki\text{1}}+\overset{\left(\text{1}\right)}{\underset{k}{bi}} \end{array}$$ (16)

$$\begin{array}{c} V_{i\text{1}}={AXi}^{\left(\text{1}\right)}\;W^{vi\text{1}}+\overset{\left(\text{1}\right)}{\underset{v}{bi}} \end{array}$$ (17)

where $W^{Qi\text{1}}$, $W^{ki\text{1}}$ and $W^{vi\text{1}}$ are the weight matrices for the query, key, and value of $i_{t\text{1}}$, respectively, while $Q_{i\text{1}}$, $K_{i\text{1}}$, and $V_{i\text{1}}$ are the corresponding query, key, and value matrices of $i_{t\text{1}}$. Since the DG-LSTM-SA does not update ${\tilde{C}}_{t}$ after each layer of input gate computation, the model introduces $\overset{\left(\text{1}\right)}{\underset{q}{bi}}$, $\overset{\left(\text{1}\right)}{\underset{k}{bi}}$ and $\overset{\left(\text{1}\right)}{\underset{v}{bi}}$ as bias terms for $Q_{i\text{1}}$, $K_{i\text{1}}$ and $V_{i\text{1}}$ to enhance the flexibility of the first layer input gate and mitigate the negative impact of delayed ${\tilde{C}}_{t}$ updates on the model’s data-fitting capability.

The core of the attention mechanism in the first layer input gate involves calculating the dot product of $Q_{i\text{1}}$ and $K_{i\text{1}}$ to obtain the attention weight $A_{i\text{1}}$. This process is accomplished by computing the similarity between $Q_{i\text{1}}$ and $K_{i\text{1}}$, followed by applying the softmax function, as described by Eq (18):


$$\begin{array}{c} A_{i\text{1}}=\text{softmax}(\frac{Q_{i\text{1}}\;{K_{i\text{1}}}^{T}}{\sqrt{d_{ki\text{1}}}}) \end{array}$$ (18)

where $Q_{i\text{1}}\;{K_{i\text{1}}}^{T}$ represents the product of the query matrix and the transposed key matrix. $d_{ki\text{1}}$ is the dimensionality of the key vectors. $A_{i\text{1}}$ is the attention weight matrix, corresponding to each element in the sequence ${AXi}^{\left(\text{1}\right)}$.

Next, each row of the matrix $A_{i\text{1}}$ is multiplied by the columns of the matrix $V_{i\text{1}}$ via a dot product operation. The output vector ${iOT}_{{t\text{1}}_{i}}$ for each element in the sequence is the weighted sum of its attention weights and all value vectors, described by Eq (19):


$$\begin{array}{c} {iOT}_{{t\text{1}}_{i}}=\sum_{j=\text{1}}^{n}{A_{i\text{1}}}_{ij}{V_{i\text{1}}}_{j} \end{array}$$ (19)

where ${A_{i\text{1}}}_{ij}$ is the element in the i th row and j th column of matrix $A_{i\text{1}}$, representing the attention weight of element $\overset{\left(\text{1}\right)}{\underset{j}{AXi}}$ on element $\overset{\left(\text{1}\right)}{\underset{i}{AXi}}$. ${V_{i\text{1}}}_{j}$ is the j th vector in $V_{i\text{1}}$. By aggregating the output vectors ${iOT}_{{t\text{1}}_{i}}$ of all elements, the final output matrix ${iOT}_{t\text{1}}=[{iOT}_{{t\text{1}}_{\text{1}}};{iOT}_{{t\text{1}}_{\text{2}}};\ldots ;{iOT}_{{t\text{1}}_{n}}]$ is formed. The matrix representing the weighted sum of $V_{i\text{1}}$ using the attention weights is given by Eq (20):


$$\begin{array}{c} {iOT}_{t\text{1}}=A_{i\text{1}}\;V_{i\text{1}}. \end{array}$$ (20)

Where ${iOT}_{t\text{1}}$ is the output information of the first layer input gate’s attention mechanism.

To further capture higher-level sequence features, ${iOT}_{t\text{2}}$ uses the output of the first layer input gate as its input, i.e., ${AXi}^{\left(\text{2}\right)}=i_{t\text{1}}$. For each element $\overset{\left(\text{2}\right)}{\underset{i}{AXi}}$ in the sequence ${AXi}^{\left(\text{2}\right)}$, it is mapped to its query, key, and value vectors using three different weight matrices $Q_{i\text{2}}$, $K_{i\text{2}}$ and $V_{i\text{2}}$, allowing the model to query different parts of the input sequence and assign attention scores to each part. In this study, we also perform a dot product operation between each row of the attention weight matrix $A_{i\text{2}}$ and the columns of the matrix $V_{i\text{2}}$, such that the output vector ${iOT}_{{t\text{2}}_{i}}$ for each element in the sequence is the weighted sum of its attention weights and all value vectors. After weighting $V_{i\text{2}}$ with the attention weights, a new representation ${iOT}_{t\text{2}}$ is obtained, which integrates different parts of the input sequence ${AXi}^{\left(\text{2}\right)}$, further enhancing the DG-LSTM-SA’s ability to capture long-term dependencies.

As shown in Fig 2B, the model integrates two-layer of self-attention mechanisms, ${iOT}_{t\text{1}}$ and ${iOT}_{t\text{2}}$, within $i_{t\text{1}}$ and $i_{t\text{2}}$, respectively, to enhance the decision-making capabilities of these input gates. As depicted in Eqs (21) and (22), ${iOT}_{t\text{1}}$ represents the sequence representation obtained by weighting the previous DG-LSTM-SA unit’s output $h_{t-\text{1}}$ and the current unit’s input $x_{t}$. ${iOT}_{t\text{1}}$ incorporates the attention information from the elements within the sequence $\left[h_{t-\text{1}},x_{t}\right]$. After reweighting ${iOT}_{t\text{1}}$, $x_{t}$ and $h_{t-\text{1}}$ using the weight $W_{i\text{1}}$, the sigmoid function is applied to further enhance the first layer input gate’s ability to filter critical information from the raw input data. ${iOT}_{t\text{2}}$ integrate the element information from sequences $i_{t\text{1}}$, to further improve the input gate’s ability to select the most useful information.


$$\begin{array}{c} i_{t\text{1}}=\sigma (W_{i\text{1}}\cdot [h_{t-\text{1}},x_{t},{iOT}_{t\text{1}}]+b_{i\text{1}}) \end{array}$$ (21)

$$\begin{array}{c} i_{t\text{2}}=\sigma (W_{i\text{2}}\cdot [i_{t\text{1}},{iOT}_{t\text{2}}]+b_{i\text{2}}) \end{array}$$ (22)

### 2.3 Deep output gated mechanism

**Fig 3:**
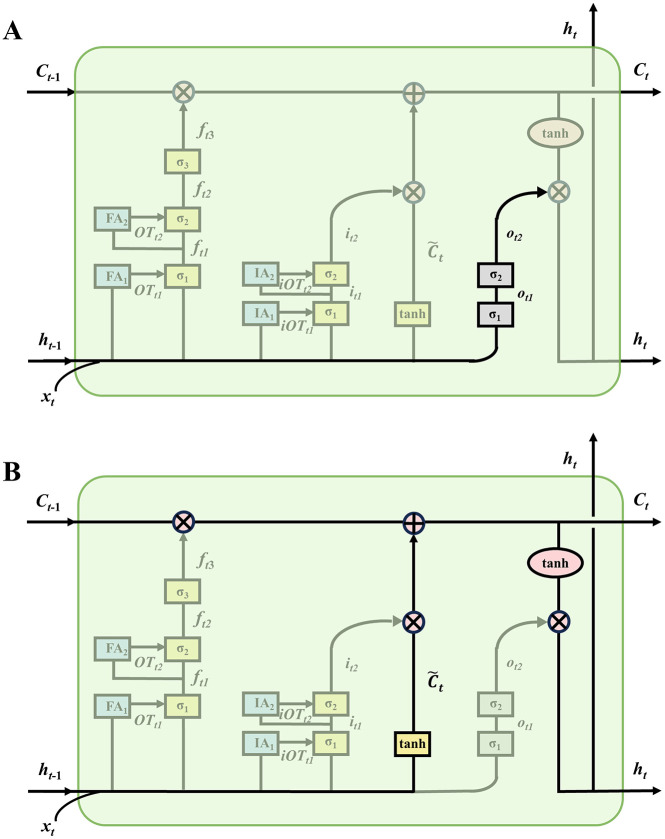
Schematic of the two-layer output gate (A) and output unit (B) in the DG-LSTM-SA model.


As shown in Fig 3A, the designed output gate has a two-layer structure. The first layer output gate is similar to the output gate in a traditional LSTM, responsible for initially filtering the important information from $h_{t-\text{1}}$ and $x_{t}$, and controlling its entry into the next layer output gate of the DG-LSTM-SA, as described in Eq (23):

$$\begin{array}{c} o_{t\text{1}}=\sigma_{\text{1}}(W_{o\text{1}}\cdot [h_{t-\text{1}},x_{t}]+b_{o\text{1}}) \end{array}$$ (23)

where $o_{t\text{1}}$ represents the first layer output gate, obtained by computing the input data $x_{t}$ of the current DG-LSTM-SA unit and the output $h_{t-\text{1}}$ from the previous unit using the output gate weight matrix $W_{o\text{1}}$, adding the bias $b_{o\text{1}}$, and then applying a sigmoid function $\sigma_{\text{1}}$.

The contextual information carried by the first layer output gate $o_{t\text{1}}$ is directly passed to the second layer output gate, where it is processed with the second layer weight matrix $W_{o\text{2}}$. The result is summed with the bias $b_{o\text{2}}$ and then passed through a sigmoid function $\sigma_{\text{2}}$ to obtain the second layer output gate $o_{t\text{2}}$, as described by Eq (24):


$$\begin{array}{c} o_{t\text{2}}=\sigma_{\text{2}}(W_{o\text{2}}\cdot o_{t\text{1}}+b_{o\text{2}}) \end{array}$$ (24)

### 2.4 Output of the DG-LSTM-SA unit

As shown in Fig 3B, the output of the DG-LSTM-SA unit mainly consists of two parts: the cell state $C_{t}$ and the hidden state $h_{t}$, which together determine the information retained and transmitted by the DG-LSTM-SA unit at the current time step. The cell state $C_{t}$ is updated in two main phases: 1) Phase of forgetting old information. The output of the forget gate $f_{t\text{3}}$ is multiplied by the cell state of the previous time step $C_{t-\text{1}}$, i.e., $f_{t\text{3}}\times C_{t-\text{1}}$. $f_{t\text{3}}$ ranges between 0 and 1; if the output of the third layer forget gate is close to 0, the forget gate closes, leading to the forgetting of information in the cell state. If the output is close to 1, the forget gate opens, and the information is retained. 2) Phase of adding new information. The sigmoid process of the two-layer input gate determines the information to be updated, $i_{t\text{2}}$, and then a candidate vector ${\tilde{C}}_{t}$ is created by the tanh layer. The output of the third layer input gate $i_{t\text{2}}$ is then multiplied by ${\tilde{C}}_{t}$, i.e., $i_{t\text{2}}\times {\tilde{C}}_{t}$, to ensure that the cell state is updated only when the input gate $i_{t\text{2}}$ is activated, as shown in Eq (25):


$$\begin{array}{c} {\tilde{C}}_{t}=\text{tanh}\left(W_{C}\cdot \left[h_{t-\text{1}},x_{t}\right]+b_{C}\right) \end{array}$$ (25)

where $W_{C}$ is the weight matrix for the candidate cell state, and $b_{C}$ is the bias term for the candidate cell state.

Finally, the new cell state ${\tilde{C}}_{t}$ is obtained by adding the product of the third layer forget gate $f_{t\text{3}}$ and the cell state $C_{t-\text{1}}$ to the product of the second layer input gate $i_{t\text{2}}$ and the candidate vector ${\tilde{C}}_{t}$, as expressed in Eq (26):


$$\begin{array}{c} C_{t}=f_{t\text{3}}⊙C_{t-\text{1}}+i_{t\text{2}}⊙{\tilde{C}}_{t} \end{array}$$ (26)

where ⊙ represents the element-wise (Hadamard) product.

After updating the cell state $C_{t}$, the cell state information controlled by the second layer output gate $o_{t\text{2}}$ is used to calculate the hidden state $h_{t}$. First, the activation of the output gate determines which parts of the cell state will be output based on the extent to which the third layer output gate $o_{t\text{2}}$ is open. Next, cell state adjustment is performed by applying the tanh function to the current cell state $C_{t}$, i.e., $\text{tanh}\left(C_{t}\right)$, to compress the data between −1 and 1, thus aiding in numerical stability. Finally, the hidden state $h_{t}$ is computed by multiplying the output $o_{t\text{2}}$ of the second layer output gate $\sigma_{\text{2}}$ with the adjusted cell state, ensuring that only the information transmitted by the output gate $o_{t\text{2}}$ serves as the output for the hidden state $h_{t}$, as shown in Eq (27):


$$\begin{array}{c} h_{t}=o_{t\text{2}}⊙\text{tanh}\left(C_{t}\right) \end{array}$$ (27)

## 3 Experimental data and evaluation

### 3.1 Experimental setup

All experiments were conducted on a workstation equipped with an NVIDIA RTX 2060 GPU and 64 GB of RAM. All models were implemented in Python 3.8 using PyTorch 1.9.0. We used stochastic gradient descent (SGD) with an initial learning rate of 0.001. Early stopping was applied to mitigate overfitting; specifically, training was terminated if the validation loss did not improve for 10 consecutive epochs. The loss function is $L(y,\hat{y})=\sum_{t=\text{1}}^{T}\;|{\hat{y}}_{t}-y_{t}\overset{\text{2}}{\underset{\text{2}}{|}}$, where $y_{t}=x_{t+\text{1}}$ and ${\hat{y}}_{t}$ denote the ground truth and prediction, respectively. Reported results are averaged over five independent runs with the same random seed to ensure reproducibility. Hyperparameters were selected via grid approach, including gate depths, attention placement, hidden size, dropout, batch size, and early-stopping settings (Table 1). The selected configuration (e.g., 3 forget layers, 2 input layers, hidden size of 128, dropout of 0.2) consistently achieved optimal performance across multiple metrics.

**Table 1:** Hyperparameters of the DG-LSTM-SA model.

Hyperparameter	Value	Grid
Forget gate layer	3	[1, 2, 3, 4, 5, 6]
Input gate layer	2	[1, 2, 3, 4, 5, 6]
Output gate layer	2	[1, 2, 3, 4, 5, 6]
Forget gate attention mechanism layer	2	[1, 2, 3]
Input gate attention mechanism layer	2	[1, 2]
Output gate attention mechanism layer	0	[1, 2]
Epochs	32	32
Batches per epoch	64	[32, 64, 128]
Hidden size	128	[16, 32, 64, 128, 256]
Dropout	0.2	[0.1, 0.2, 0.3]
Parameter optimization	SGD	SGD
Initial learning rate	0.001	0.001
Learning rate decay	0.5	0.5
Overfitting termination	10	[6, 8, 10, 12]

### 3.2 Dataset and analysis

To validate DG-LSTM-SA on energy forecasting tasks, we conducted comparative experiments on three public datasets, including NEPOOL [29], Yichang [34], and Solar-Energy [32]. DG-LSTM-SA was compared against ten baseline models: LSTM [18], GRU [35], RNN [36], SKIP-RNN [23], IndRNN [22], LSTM-g [24], xLSTM [25], Transformer [28], Crossformer [30], and Informer [29]. Baseline hyperparameters are summarized in Table 2. The NEPOOL dataset contains hourly average load data from the New England power market. The Yichang dataset provides electricity load measurements from Yichang (China) sampled every 15 minutes over 14 months. The Solar-Energy dataset contains solar power generation records from 137 photovoltaic stations in Alabama sampled every 10 minutes in 2006. Data statistics for the three datasets, comprising power production, load, and consumption records, are summarized in Table 3.

**Table 2:** Hyperparameters of the baseline models.

RNN family (LSTM, GRU, RNN, SKIP-RNN, IndRNN, LSTM-g, xLSTM)		Transformer family (Transformer, Crossformer, Informer)	
Hidden size	128	Embedding dimension	128
Hidden layers number	4	Feed-forward dimension	512
Sequence length	100	Attention heads	8
Batch size	64	Encoder layers	6
Learning rate	0.001	Batch size	64
Optimizer	SGD	Learning rate	0.001
Dropout	0.1	Optimizer	AdamW
Layer normalization	Enabled	Attention dropout	0.1

**Table 3:** Data statistics for the three datasets.

Datasets	Total number	Training set	Validation set	Test set
NEPOOL	61281	35064	8673	17544
Yichang	38016	26611	3802	7603
Solar-Energy	7204720	5043294	720472	1440944

### 3.3 Model evaluation metrics

This study employs a multi-faceted evaluation framework comprising Mean Absolute Error (MAE), Mean Squared Error (MSE), symmetric Mean Absolute Percentage Error (sMAPE), and Root Mean Square Error (RMSE). MAE quantifies average absolute deviation, providing robust baseline assessment [32]. MSE emphasizes large errors critical for grid stability [37]. sMAPE enables scale-free comparison across heterogeneous datasets [38]. RMSE preserves MSE’s outlier sensitivity while maintaining interpretability in physical units [32]. Their calculation can be expressed by Eqs (28)–(31):


$$\begin{array}{c} \text{MSE}=\frac{\text{1}}{n}\sum_{i=\text{1}}^{n}\;(\overset{\left(i\right)}{\underset{test}{y}}-\overset{\left(i\right)}{\underset{test}{\hat{y}}}{)}^{\text{2}} \end{array}$$ (28)

$$\begin{array}{c} \text{MAE}=\frac{\text{1}}{n}\sum_{i=\text{1}}^{n}\;|\overset{\left(i\right)}{\underset{test}{y}}-\overset{\left(i\right)}{\underset{test}{\hat{y}}}| \end{array}$$ (29)

$$\begin{array}{c} \text{sMAPE}=\frac{\text{1}\text{0}\text{0}\%}{n}\sum_{i=\text{1}}^{n}\frac{\text{2}|\overset{\left(i\right)}{\underset{test}{y}}-\overset{\left(i\right)}{\underset{test}{\hat{y}}}|}{\left|\overset{\left(i\right)}{\underset{test}{y}}\right|+\left|\overset{\left(i\right)}{\underset{test}{\hat{y}}}\right|} \end{array}$$ (30)

$$\begin{array}{c} \text{RMSE}=\sqrt{\frac{\text{1}}{n}\sum_{i=\text{1}}^{n}\;(\overset{\left(i\right)}{\underset{test}{y}}-\overset{\left(i\right)}{\underset{test}{\hat{y}}}{)}^{\text{2}}} \end{array}$$ (31)

where n represents the number of samples in the test set; $\overset{\left(i\right)}{\underset{test}{y}}$ denotes the i-th actual observed value in the test set, and $\overset{\left(i\right)}{\underset{test}{\hat{y}}}$ represents the i-th predicted value. $(\overset{\left(i\right)}{\underset{test}{y}}-\overset{\left(i\right)}{\underset{test}{\hat{y}}}{)}^{\text{2}}$ represents the square of the difference between the actual and predicted values, used to eliminate the positive and negative effects of errors and to assign greater weight to larger errors, while $\left|\overset{\left(i\right)}{\underset{test}{y}}-\overset{\left(i\right)}{\underset{test}{\hat{y}}}\right|$ assigns equal weight to all errors.

## 4 Results and discussion

### 4.1 Optimization of the DG-LSTM-SA model

**Fig 4:**
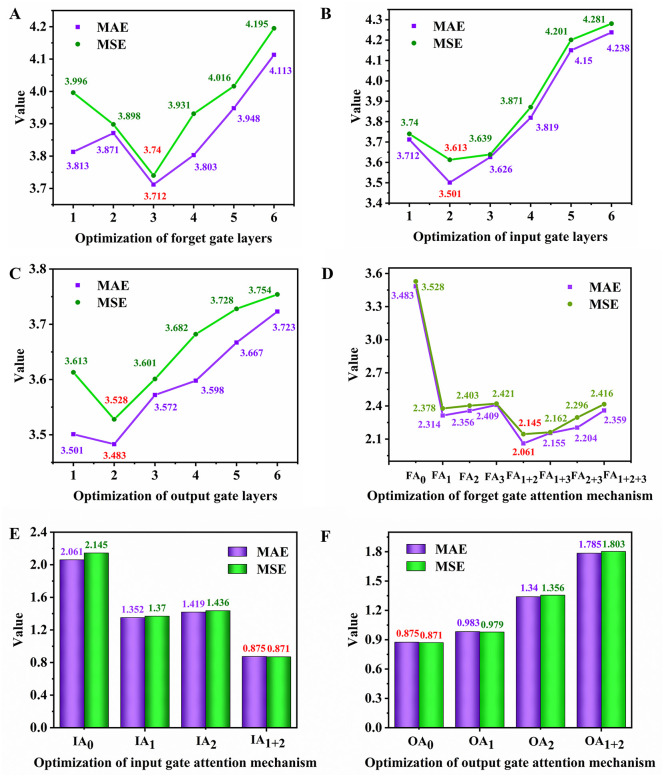
Optimization of forget gate layers (A), input gate layers (B), output gate layers (C), forget gate attention mechanism (D), input gate attention mechanism (E), and output gate attention mechanism.

To obtain the best-performing DG-LSTM-SA configuration, we first optimized the number of layers for the forget, input, and output gates on the NEPOOL dataset, and then optimized the placement (and number) of self-attention modules (Fig 4). MAE and MSE were used as primary criteria. When increasing the forget-gate depth from 1 to 6 while keeping other components consistent with a baseline LSTM, the best performance was achieved with three forget-gate layers (MAE = 3.712, MSE = 3.740; Fig 4A). Further increasing the number of layers led to performance degradation, indicating that a three-layer structure achieves a balance between capturing long-term dependencies and avoiding overfitting. With the forget gate fixed at three layers, the input-gate depth was tuned from 1 to 6, and a two-layer input gate achieved the best results (MAE = 3.501, MSE = 3.613; Fig 4B). Finally, with the forget and input gates fixed at 3 and 2 layers, respectively, the best output-gate depth was two layers (MAE = 3.483, MSE = 3.528; Fig 4C).

To further enhance prediction performance, we then introduced self-attention to improve feature screening while avoiding excessive parameter growth. Applying attention to the first two forget-gate layers yielded the best performance (MAE = 2.061, MSE = 2.145; Fig 4D). After optimizing the forget gate attention, we optimized attention in the input gate; applying attention to both input-gate layers performed best (MAE = 0.875, MSE = 0.871; Fig 4E). In contrast, introducing attention into the output gate reduced accuracy (Fig 4F), indicating that the output gate already provides sufficient filtering and additional attention may destabilize the output mapping. Therefore, the final configuration adopts 3/2/2 layers for the forget/input/output gates, respectively, and applies attention to the first two layers of both the forget and input gates (Fig 1A).

### 4.2. Evaluation of time complexity and training efficiency

To assess practical deployability, we compared DG-LSTM-SA with mainstream forecasting models in terms of theoretical time complexity and measured training efficiency (seconds per epoch, s/epoch). As shown in Table 4, Transformer-based models typically scale quadratically (or worse) with sequence length, leading to higher computational cost. DG-LSTM-SA introduces additional gating and attention operations, but its overall complexity remains comparable to LSTM-based variants and is lower than global-attention models. Moreover, the hierarchical design reduces redundant computations.

**Table 4:** Time complexity of the DG-LSTM-SA and baseline models.

Models	Time complexity
DG-LSTM-SA	$\text{O}(\text{7}\overset{\text{2}}{\underset{h}{n}}+\left(\text{7}n_{in}+\text{12}d_{a}\right){\cdot n}_{h}+\text{4}d_{a}\cdot \text{T})$
RNN	$\text{O}(\text{T}\cdot (n_{in}\cdot n_{h}+\overset{\text{2}}{\underset{h}{n}}))$
GRU	$\text{O}(\text{3T}\cdot (n_{in}\cdot n_{h}+\overset{\text{2}}{\underset{h}{n}}))$
LSTM	$\text{O}(\text{4T}\cdot (n_{in}\cdot n_{h}+\overset{\text{2}}{\underset{h}{n}}))$
LSTM-g	$\text{O}(\text{6T}\cdot (n_{in}\cdot n_{h}+\overset{\text{2}}{\underset{h}{n}}))$
IndRNN	$\text{O}(\text{T}\cdot \overset{\text{2}}{\underset{h}{n}})$
SKIP-RNN	$\text{O}(\text{kT}\cdot (n_{in}\cdot n_{h}+\overset{\text{2}}{\underset{h}{n}}))$
xLSTM	$\text{O}(\text{T}\cdot (\text{7}n_{in}\cdot n_{h}+\text{8}\overset{\text{2}}{\underset{h}{n}}))$
Transformer	$\text{O}({\text{T}}^{\text{2}}\cdot d_{model}+\text{T}\cdot \overset{\text{2}}{\underset{model}{d}})$
Informer	$\text{O}(\text{T}\cdot \text{logT}\cdot d_{model})$
Crossformer	$\text{O}({\text{L}}^{\text{2}}\cdot {\text{T}}^{\text{1}+\text{1}/\text{L}}\cdot d_{model}+{\text{L}}^{\text{2}}\cdot \text{T}\cdot \overset{\text{1}+\text{1}/\text{L}}{\underset{model}{d}})$

$n_{h}$, Hidden size; $n_{in}$, Input dimension; $d_{a}$: Attention dimension; T, Sequence length; k, Skipping coefficient; $d_{model}$, Embedding dimension; L, Cross-scale hierarchy levels.

**Fig 5:**
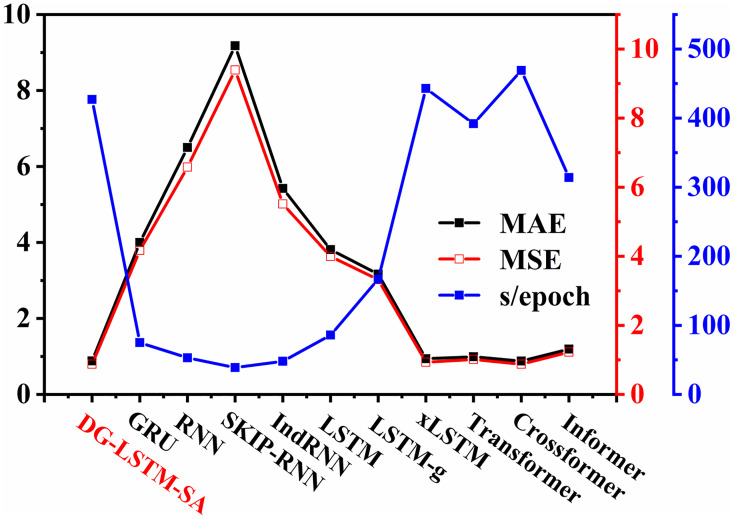
Prediction accuracy and training efficiency of the DG-LSTM-SA and baseline models.

Moreover, we further compared the predictive performance and training efficiency of DG-LSTM-SA against other baseline models. As illustrated in Fig 5, DG-LSTM-SA achieves the lower MAE (0.883) and MSE (0.878) values, comparable to Crossformer (MAE = 0.881, MSE = 0.877), while training faster (427 s/epoch vs. 469 s/epoch). Compared with xLSTM (443 s/epoch), DG-LSTM-SA provides better accuracy with competitive efficiency. Conventional RNN variants train faster but yield substantially higher errors. Overall, DG-LSTM-SA offers a strong balance between forecasting accuracy and computational efficiency, making it suitable for real-world grid scheduling applications.

### 4.3 Prediction performance on power generation and load datasets

#### 4.3.1 Power generation dataset (solar-energy).

**Fig 6:**
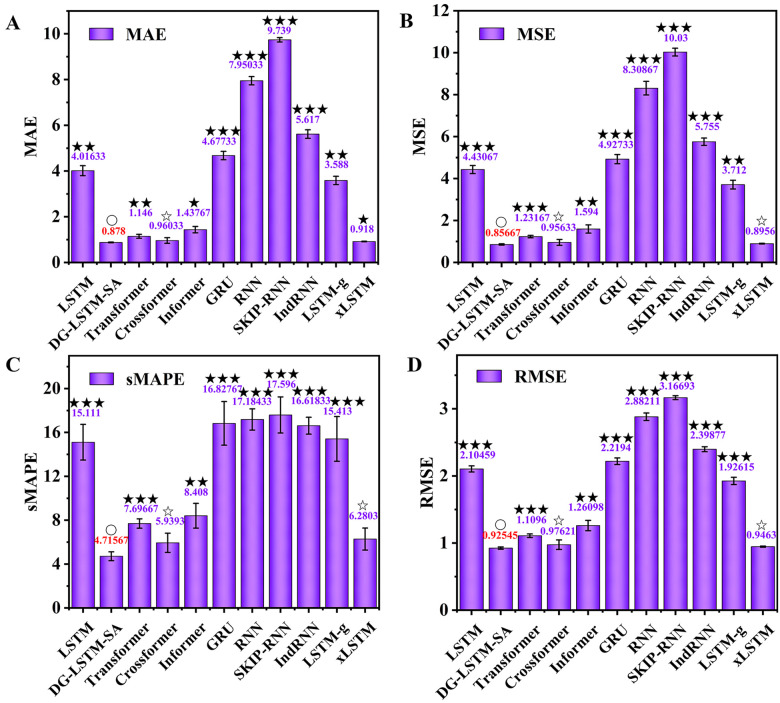
Comparison of the DG-LSTM-SA model and ten baseline models on Solar-Energy dataset. **(A)** MAE; **(B)** MSE; (C) sMAPE; **(D)** RMSE. Significance between the DG-LSTM-SA and ten baseline models was analyzed using the *t*-test. The symbol ‘○’ denotes control group, ‘☆’ denotes P > 0.05, ‘★’ denotes P < 0.05, ‘★★’ denotes P < 0.01, ‘★★★’ denotes P < 0.001.

To comprehensively evaluate the predictive performance, we compared DG-LSTM-SA with ten baselines on Solar-Energy using MAE, MSE, sMAPE, and RMSE. As shown in Fig 6, DG-LSTM-SA model achieved the best overall performance (MAE = 0.878, MSE = 0.857, sMAPE = 4.716%, RMSE = 0.925). Compared with LSTM, DG-LSTM-SA reduced MAE by 3.138 (P < 0.01), MSE by 3.574 (P < 0.001), sMAPE by 10.395% (P < 0.001), and RMSE by 1.179 (P < 0.001). Similarly, relative to GRU architecture, DG-LSTM-SA reduced MAE by a factor of 5.327 (P < 0.001) and achieved consistent improvements across the other metrics (Fig 6). In terms of sMAPE, which provides a scale-independent measure of accuracy, DG-LSTM-SA significantly outperformed all benchmarks. Its sMAPE value of 4.716% was less than one-third that of SKIP-RNN (17.596%, P < 0.001) and RNN (17.184%, P < 0.001), indicating superior relative prediction accuracy (Fig 6C). Moreover, the proposed model achieved the lowest RMSE of 0.925 (Fig 6D), reflecting the smallest standard deviation of prediction residuals and thus the highest overall prediction consistency. The substantial improvement over traditional recurrent models can be attributed to the structural limitations of standard RNNs. Solar energy generation is highly volatile and heavily influenced by sudden meteorological changes. Standard LSTMs and GRUs rely solely on a compressed hidden state to carry historical context, causing an information bottleneck when handling long sequences with sudden spikes. In contrast, the hierarchical self-attention embedded in the DG-LSTM-SA dynamically screens and assigns higher weights to critical historical fluctuations, allowing the model to adaptively capture sudden weather-driven changes without losing long-term trend information.

Among recent state-of-the-art models, DG-LSTM-SA also demonstrated superior accuracy (Fig 6). It outperformed Informer, with a 38.94% reduction in MAE (P < 0.05, Fig 6A), 46.24% in MSE (P < 0.01, Fig 6B), 43.91% in sMAPE (P < 0.01, Fig 6C), and 36.32% in RMSE (P < 0.01, Fig 6D), and exceeded Transformer by 30.52% in MAE (P < 0.01, Fig 6A), 43.76% in MSE (P < 0.001, Fig 6B), 63.21% in sMAPE (P < 0.001, Fig 6C), and 16.67% in RMSE (P < 0.001, Fig 6D). Furthermore, while Crossformer and xLSTM showed competitive results, their MAE values were 9.34% (P > 0.05) and 4.56% (P < 0.05) higher (Fig 6A), than those of DG-LSTM-SA, with corresponding increases in RMSE of 5.51% (P > 0.05) and 2.27% (P > 0.05) (Fig 6D). The results confirm that the proposed model not only significantly surpasses traditional RNN-based architectures but also maintains an advantage over contemporary attention-based models in both absolute and relative error metrics, highlighting its strong capability in handling complex multi-periodic patterns in solar energy data. These comparative results well support our initial hypothesis discussed in the introduction. While traditional recurrent models often struggle to adaptively weight volatile time steps [22–25] and recent Transformer variants tend to introduce excessive computational overhead [28–30], the proposed DG-LSTM-SA architecture successfully mitigates both issues by organically integrating deep gating structures with internal self-attention.

#### 4.3.2 Analysis of power load datasets.

**Fig 7:**
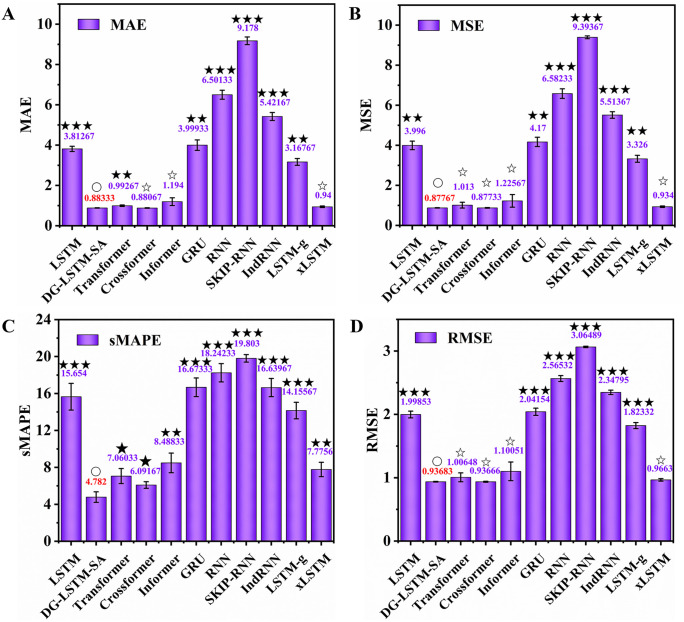
Comparison of the DG-LSTM-SA model and ten baseline models on NEPOOL dataset. **(A)** MAE; **(B)** MSE; (C) sMAPE; **(D)** RMSE. Significance between the DG-LSTM-SA and ten baseline models was analyzed using the *t*-test. The symbol ‘○’ denotes control group, ‘☆’ denotes P > 0.05, ‘★’ denotes P < 0.05, ‘★★’ denotes P < 0.01, ‘★★★’ denotes P < 0.001.

To further validate the generalization capability of the DG-LSTM-SA model, we extended the comparative experiments to two power load forecasting datasets, NEPOOL and Yichang, using the same ten baseline models and evaluation metrics. As shown in Fig 7, on the NEPOOL dataset, the proposed DG-LSTM-SA model achieved highly competitive performance across all metrics, with an MAE of 0.883, MSE of 0.878, sMAPE of 4.782%, and RMSE of 0.937. Although Crossformer also performed strongly (MAE = 0.881, P > 0.05; MSE = 0.877, P > 0.05), DG-LSTM-SA maintained comparable prediction accuracy while demonstrating better consistency in error distribution (Fig 7A and 7B). Compared to traditional models, the improvements were substantial: MAE was reduced by 86.42% over RNN (6.501, P < 0.001), 90.38% over SKIP-RNN (9.178, P < 0.001), 76.84% over LSTM (3.813, P < 0.001), and 77.92% over GRU (3.999, P < 0.01) (Fig 7A), while sMAPE observed a decrease of 73.79% over RNN (18.24%, P < 0.001), 75.85% over SKIP-RNN (19.80%, P < 0.001), 69.45% over LSTM (15.65%, P < 0.001), and 71.33% over GRU (16.67%, P < 0.001) (Fig 7C). The model also outperformed recent architectures such as xLSTM (sMAPE = 7.776%, P < 0.01), Crossformer (sMAPE = 6.092%, P < 0.05), and Transformer (sMAPE = 7.060%, P < 0.05) (Fig 7C).

**Fig 8:**
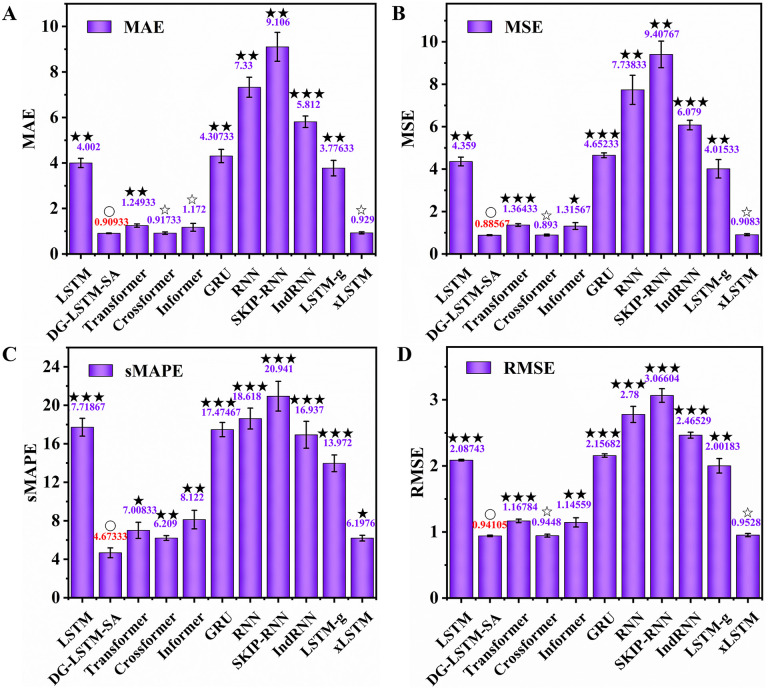
Comparison of the DG-LSTM-SA model and ten baseline models on Yichang dataset. **(A)** MAE; **(B)** MSE; (C) sMAPE; **(D)** RMSE. Significance between the DG-LSTM-SA and ten baseline models was analyzed using the *t*-test. The symbol ‘○’ denotes control group, ‘☆’ denotes P > 0.05, ‘★’ denotes P < 0.05, ‘★★’ denotes P < 0.01, ‘★★★’ denotes P < 0.001.

On the Yichang dataset, DG-LSTM-SA again delivered the best overall results, attaining an MAE of 0.909, MSE of 0.886, sMAPE of 4.673%, and RMSE of 0.941 (Fig 8). It consistently surpassed all other models across every metric. Notably, it improved upon Crossformer (MAE = 0.917, MSE = 0.893) by approximately 0.87% in MAE and 0.78% in MSE (Fig 8A and 8B), and significantly outperformed Transformer (MSE = 1.364, P < 0.001; RMSE = 1.168, P < 0.001; Fig 8B and 8D). Compared to LSTM (MAE = 4.002) and GRU (MAE = 4.307), the proposed model reduced MAE by over 77.29% (P < 0.001) and 78.89% (P < 0.001), underscoring its effectiveness in capturing complex load patterns (Fig 8A). Furthermore, the sMAPE value of 4.673% was the lowest among all models, indicating superior relative prediction accuracy, particularly when compared to IndRNN (16.937%, P < 0.001) and LSTM-g (13.972%, P < 0.001) (Fig 8C). The combined results across both power load datasets confirm that DG-LSTM-SA not only achieves state-of-the-art forecasting accuracy but also exhibits strong generalization across different grid systems. Its ability to consistently outperform both classical RNN variants and modern attention-based models highlights its robustness and practical utility in real-world load forecasting scenarios. Power load datasets generally exhibit strong multi-scale periodicities (e.g., daily and weekly cycles) mixed with short-term stochastic load behaviors. Transformer-based models, while powerful, inherently lack a sequential inductive bias, which forces them to rely on heavy positional encodings that sometimes fail to capture strict temporal ordering. The proposed DG-LSTM-SA overcomes this by retaining the step-by-step recurrence of LSTMs while utilizing deep multi-layer gating to filter out noise across different temporal scales. This hybrid mechanism ensures that the structural integrity of periodic load patterns is maintained, while the attention modules capture anomalous consumption behaviors, leading to superior generalization on both the NEPOOL and Yichang datasets.

### 4.4 Ablation study of the DG-LSTM-SA model

To thoroughly evaluate the contribution of each component in the proposed DG-LSTM-SA architecture, a comprehensive ablation study was conducted. As shown in Fig 1, the full model consists of three forget gate layers (F1, F2, F3), two input gate layers (I1, I2), two output gate layers (O1, O2), with self-attention mechanisms applied to the first and second layers of both the forget gate (FA1, FA2) and the input gate (IA1, IA2). We systematically ablated these components and evaluated the impact on performance using MAE and MSE, as summarized in Table 5. The complete model (all components enabled) achieved the optimal performance, with an MAE of 0.883 and MSE of 0.878. Ablating the self-attention mechanism on the second layer of the input gate (IA2) resulted in an MAE of 1.352 and MSE of 1.370, while removing the self-attention on the first input gate layer (IA1) further increased MAE to 1.419 and MSE to 1.436. Disabling both input gate attention modules (IA1 and IA2) led to a more significant performance drop, with MAE and MSE rising to 2.061 and 2.145, respectively. These results highlight the critical role of the self-attention mechanism in the input gate for feature filtering. Subsequently, removing the self-attention from the second forget gate layer (FA2) increased MAE to 2.314 and MSE to 2.378. When the attention on the first forget gate layer (FA1) was also ablated, performance further decreased (MAE = 2.356, MSE = 2.403). Eliminating all attention mechanisms across both gates resulted in even higher errors (MAE = 3.483, MSE = 3.528), underscoring the importance of attention in capturing long-term dependencies and refining historical information.

**Table 5:** Effect of different component ablation on model performance.

Forget gate			Self-attention module		Input gate		Self-attention module		Output gate		MAE	MSE
F1	F2	F3	FA1	FA2	I1	I2	IA1	IA2	O1	O2		
√	√	√	√	√	√	√	√	√	√	√	**0.883** ^a^	**0.878** ^a^
√	√	√	√	√	√	√	√	✕	√	√	1.352	1.370
√	√	√	√	√	√	√	✕	√	√	√	1.419	1.436
√	√	√	√	√	√	√	✕	✕	√	√	2.061	2.145
√	√	√	√	✕	√	√	✕	✕	√	√	2.314	2.378
√	√	√	✕	√	√	√	✕	✕	√	√	2.356	2.403
√	√	√	✕	✕	√	√	✕	✕	√	√	3.483	3.528
√	√	√	✕	✕	√	√	✕	✕	√	✕	3.501	3.613
√	√	√	✕	✕	√	✕	✕	✕	√	✕	3.712	3.740
√	√	✕	✕	✕	√	✕	✕	✕	√	✕	3.871	3.898
√	✕	✕	✕	✕	√	✕	✕	✕	√	✕	3.813	3.996

^a^**Bold** indicates the optimal MAE and MSE values; F1, F2, and F3 represent the first, second, and third forget gate; FA1 and FA2 represent the first and second self-attention module on forget gate; I1 and I2 represent the first and second input gate; IA1 and IA2 represent the first and second self-attention module on input gate; O1 and O2 represent the first and second output gate.

We further investigated the impact of the deep gating structure (Table 5). Reducing the output gate from two layers to one (removing O2) increased MAE to 3.501 and MSE to 3.613. Simplifying the input gate to a single layer (removing I2) while keeping a single-layer output gate led to MAE = 3.712 and MSE = 3.740. Additional removal of the third forget gate layer (F3) further degraded performance (MAE = 3.871, MSE = 3.898). Finally, using only the first forget gate layer (F1) without any attention or advanced gating resulted in the poorest performance (MAE = 3.813, MSE = 3.996), close to that of a standard LSTM. The ablation study clearly demonstrates that both the multi-level gating design and the hierarchical self-attention mechanisms are vital to the model’s performance. The attention modules, particularly those in the lower-level gates, play an essential role in enhancing the model’s ability to dynamically weight features and temporal dependencies. Moreover, the deep gating architecture provides necessary depth for modeling complex sequences without introducing redundancy, striking an optimal balance between representation capacity and computational efficiency.

### 4.5 Comprehensive discussion on DG-LSTM-SA model

The systematic evaluations and ablation studies conducted in this research highlight several key findings regarding deep sequence modeling for energy systems. First, from a methodological perspective, our results demonstrate that completely abandoning recurrent architectures in favor of global self-attention (as seen in Transformer variants) may not be the optimal solution for energy time-series. Global attention often introduces significant computational redundancy by calculating dependencies between all time steps, many of which are physically irrelevant in power forecasting. By embedding self-attention strictly within the internal multi-layer gates of the LSTM, the DG-LSTM-SA effectively acts as a localized, dynamic filter. It explicitly forces the network to focus on essential features before updating the memory cell, thereby resolving the gradient decay issues of traditional RNNs while avoiding the 𝑂(𝑇2) complexity parameter explosion of standard Transformers.

Second, from a data characteristic perspective, the superiority of the proposed model is deeply tied to the specific nature of power generation and load data. Energy series are typically characterized by a mixture of long-term deterministic seasonality and short-term stochastic volatility. Our hierarchical gating structure effectively captures the long-term determinism by selectively passing long-range memory, while the gate-level self-attention swiftly reacts to short-term stochastic spikes (such as sudden solar irradiance drops or unexpected load peaks).

Finally, regarding practical implications, modern smart grids increasingly require decentralized decision-making and edge computing. State-of-the-art models like Informer and Crossformer, despite their high accuracy, demand substantial computational resources, limiting their deployment on resource-constrained field devices. As evidenced by our time complexity and training efficiency analysis (Sect 4.2), DG-LSTM-SA achieves matching or superior predictive precision with significantly lower training overhead. This computational efficiency, combined with high robustness against volatile data, makes the proposed model a highly practical and scalable solution for real-time power grid scheduling, dynamic resource allocation, and anomaly detection in modern energy management systems.

## 5. Conclusion

This study proposes the DG-LSTM-SA model to bridge the gap between the limited temporal adaptability of traditional recurrent networks and the excessive computational overhead of Transformers. By embedding self-attention within a deep gating structure, the model achieves highly efficient dynamic feature screening. Evaluations across three energy datasets demonstrate that DG-LSTM-SA outperforms ten baseline models, reducing the Mean Absolute Error by over 75% compared to standard LSTMs while matching the accuracy of advanced Transformer variants. Crucially, this hybrid design maintains low computational complexity and fast training speeds, proving its practicality for real-world grid dispatch. Future work will explore integrating multi-source meteorological data to further enhance forecasting reliability. Additionally, optimizing the model for resource-constrained edge devices and evaluating its resilience under extreme weather events remain key directions for advancing decentralized smart grid management.
